# Supplementary material for: Regulatory Effect of Methylation of the Porcine AQP3 Gene Promoter Region on Its Expression Level and Porcine Epidemic Diarrhea Virus Resistance
Source: Genes (Basel). 2020 Oct 6;11(10):1167. doi: 10.3390/genes11101167 (PMC7599489; doi:10.3390/genes11101167)
Supplement: Supplementary file 1 [file genes-11-01167-s001.pdf]

Regulatory Effect of Methylation of the Porcine *AQP3* Gene Promoter Region on Its Expression Level and Porcine Epidemic Diarrhea Virus Resistance

Jia-Yun Wu [1], Fang Wang [1], Zheng-Chang Wu [1,2], Sheng-Long Wu [1,2] and Wen-Bin Bao [1,2,*]

[1] Key Laboratory for Animal Genetics, Breeding, Reproduction and Molecular Design of Jiangsu Province, College of Animal Science and Technology, Yangzhou University, Yangzhou 225009, P. R. China; YZUwujiayun@163.com (J.W.); wangfangRD@163.com (F.W.); zcwu@yzu.edu.cn (Z.W.)

[2] Joint International Research Laboratory of Agriculture & Agri-Product Safety, Yangzhou University, Yangzhou, Jiangsu, P. R. China; slwu@yzu.edu.cn (S.W.)

* Correspondence: wbbao@yzu.edu.cn, Tel. : +86-514-87979316

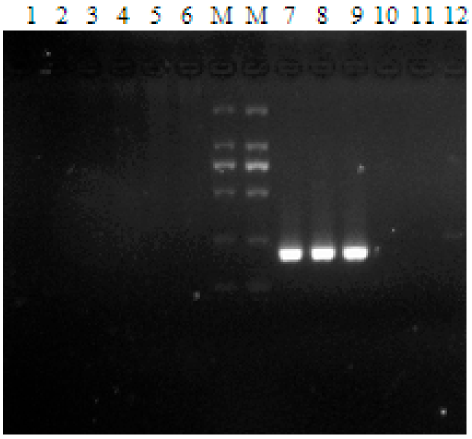

Figure S1. Agarose gel (1%) electrophoresis of PCR products amplified from the virus genome. Lanes 1–3, TGEV amplification products from jejunal tissues, jejunal contents and feces of diarrheal piglets, respectively; Lanes 4–6, PoRV amplification products; Lanes 7–9, PEDV amplification products; Lanes 10–12, mixed virus amplification products from jejunal tissues, jejunal contents and feces of normal piglets; M represents the 50-bp DNA ladder.

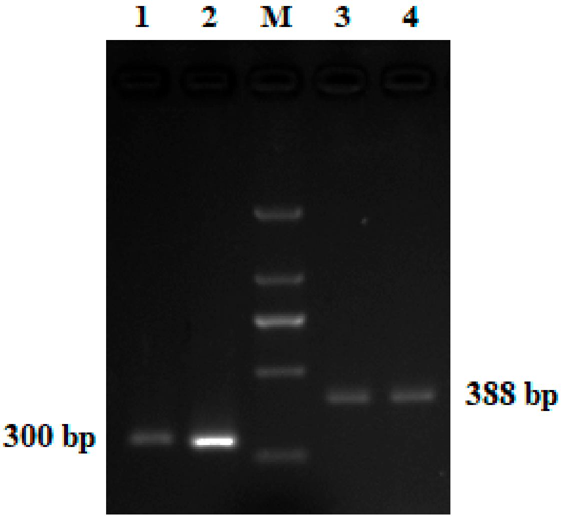

Figure S2. Amplification products of CpG islands primers. Lanes 1–2, amplification product of CpG1 primers; Lanes 3–4, amplification products of CpG2 primers. M represents the DL2000 marker.

Table S1. Information of related primers

Name	Accession number	Primer sequences
PEDV	AF353511.1	F: 5'-AGGTCTGCATTCCAGTGCTT-3'<br>R: 5'-GGACATAGAAAGCCCAACCA-3'
TGEV	FJ755618	F: 5'-CAACCCTGAAACTAACGCAATTCT-3'<br>R: 5'-GCCCATCCAGTCGCACTACTT-3'
PoRV	FJ807867	F: 5'-ATCGGGTGTCTGACTACTG-3'<br>R: 5'-TTTACTCGCATCATCCTCT-3'
AQP3	HQ888860.1	F: 5'-TCGTGATGTTTGGCTGTGG-3'<br>R: 5'-GCCAGGTTGATGGTGAGGA-3'
Sp1	XM_005652567.3	F: 5'-GTTCGCTTGCCTCGTCAGC-3'<br>R: 5'-AAAGGCACCACCATTACCACT-3'
GAPDH	AF017079.1	F: 5'-ACATCATCCCTGCTTCTACTGG -3'<br>R: 5'-CTCGGACGCCTGCTTCAC-3'
β-actin	XM_00312428.3	F: 5'-TGGCGCGCGCGCGATGAAG-3'<br>R: 5'-ACTGGACTGGCTCGT-3'
CpG1	HQ888860.1	F: 5'-TTGGAGTTTATTTTTGATAG-3'<br>R: 5'-TTATCTTTTTACCATTTATTAAACC-3'
CpG2	HQ888860.1	F: 5'-GGGTTTAGTTTTAGTTAGGTTTTTTTTTT-3'<br>R: 5'-CCCCAAACACTCAACCAATAC-3'

Table S2. Information of ChIP-PCR primers

Name	Primer sequences	Length of products (bp)
ChIP-1	F: 5'- ATTCAACCCCTAGCCTGG-3',<br>R: 5'-CATTTATTGGGCCGCTCC-3'	55
ChIP-2	F: 5'-TAGCCCCTAGGCGAGCCG -3',<br>5'-GGGTGGCGCCCTTTATAGCA-3'	100

Table S3. shRNA sequences of Sp1 interference and negative control

Name	shRNA sequences
Sp1-310	GCGGCAAAGTATATGGCAAGA
Sp1-484	GCCCTAAGCGTTTCATAAGGA
Sp1-521	GCATATCAAGACTCACCAGAA
Sp1-660	GCCATCTGTCCAGAGGGTATT
Sp1-LV3	TTCTCCGAACGTGTCACGT
